# GraphML-SBGN bidirectional converter for metabolic networks

**DOI:** 10.1515/jib-2022-0030

**Published:** 2022-12-26

**Authors:** Irina Balaur, Ludovic Roy, Vasundra Touré, Alexander Mazein, Charles Auffray

**Affiliations:** European Institute for Systems Biology and Medicine, CIRI UMR5308, CNRS-ENS-UCBL-INSERM, Université de Lyon, 50 Avenue Tony Garnier, 69007 Lyon, France; Luxembourg Centre for Systems Biomedicine, University of Luxembourg, 6 Avenue du Swing, L-4367 Belvaux, Luxembourg; Department of Biology, Norwegian University of Science and Technology (NTNU), Høgskoleringen 5, Realfagbygget, 7491 Trondheim, Norway; Russian Academy of Sciences, Institute of Cell Biophysics, 3 Institutskaya Street, Pushchino, Moscow Region, 142290, Russia

**Keywords:** GraphML, process description, Recon2 human metabolic reconstruction, systems biology graphical notation, systems biology standard

## Abstract

Systems biology researchers need feasible solutions for editing and visualisation of large biological diagrams. Here, we present the ySBGN bidirectional converter that translates metabolic pathways, developed in the general-purpose yEd Graph Editor (using the GraphML format) into the Systems Biology Graphical Notation Markup Language (SBGN-ML) standard format and vice versa. We illustrate the functionality of this converter by applying it to the translation of the ReconMap resource (available in the SBGN-ML format) to the yEd-specific GraphML and back. The ySBGN tool makes possible to draw extensive metabolic diagrams in a powerful general-purpose graph editor while providing results in the standard SBGN format.

## 1 Introduction

Systems biology standards have been developed to permit sustainability, consistency, and interoperability of biological networks and of models describing biological systems [1]. The System Biology Graphical Notation (SBGN) is the standard for biological network visualisation [2]. The Process Description (PD) is one of the three SBGN languages (in addition to Activity Flow (AF) and Entity Relationship (ER)) used for describing biochemical processes in biological systems [3].

While specialised software tools are available for the management (creation, modification) of the SBGN diagrams (http://sbgn.org/software_support/), they currently cannot compete with well-established general-purpose graph editors in terms of flexibility and usability. Consequently, there are situations when researchers use such editors to draw aesthetical diagrams (for publications or other forms of dissemination) and SBGN-specific tools to store biological content in a standard format. There is still a demand for an intuitive tool that would support drawing extensive diagrams with thousands of nodes and interconnecting edges. A brief comparison on the software that we used in several projects for editing SBGN PD maps is available in the ySBGN GitHub Wiki at https://github.com/sbgn/ySBGN/wiki/Editors [accessed May 2022]. The motivation for this work was the need of a stable environment that had the following combination of key features: (1) drawing directly in SBGN; (2) reliable support for large-scale biological maps; and (3) export to SBGN-ML. For that end, we considered to offer an SBGN converter for the general-purpose yEd Graph Editor (https://www.yworks.com/products/yed), which is a freely available tool shown to be useful for representing biological networks [4–7].

The yEd Graph Editor offers: (i) a dedicated SBGN palette (see Table 1 in *Methods* for details); (ii) an intuitive interface and snap-to-line drawing guides that significantly help the user during manual development of a network; (iii) advanced automatic layout algorithms for both generic and SBGN PD diagrams [7]; and (iv) support for producing aesthetic diagrams with the ability to make curved connectors and to easily modify colours of the elements (both edges and nodes), individually or collectively. Extended information such as annotation of the SBGN elements can be added easily as properties of the elements in yEd.

**Table 1:** SBGN PD elements and their representation in the yEd SBGN Paletteen.

SBGN element	Representation in the yEd SBGN Palette	Encoding in the yEd GraphML format
Compartment	The compartment shape	y:GroupNode
Complex	The complex shape	com.yworks.sbgn.Complex
Macromolecule	The macromolecule shape	com.yworks.sbgn.Macromolecule
Multimer simple chemical	The simple chemical multimer shape	The multimer variants of the simple chemical,
Multimer macromolecule	The macromolecule multimer shape	macromolecule, complex, nucleic acid feature classes
Multimer complex	The complex multimer shape	are denoted using the “com.yworks.sbgn.style.mcount”
Multimer nucleic acid feature	The nucleic acid feature shape	attribute attached to the main class.
Nucleic acid feature	The nucleic acid feature shape	com.yworks.sbgn.NucleicAcidFeature
AND	The AND operator shape	com.yworks.sbgn.Operator
		The SBGN operator class is
NOT	The NOT operator shape	stored as the node label in
OR	The OR operator shape	yEd SBGN Palette.
Omitted process	The omitted process shape	A regular process shape with double backslashes as its label
Perturbing agent	The perturbing agent shape	com.yworks.sbgn.PerturbingAgent
Phenotype	The phenotype shape	com.yworks.sbgn.Phenotype
Process	The process shape	com.yworks.sbgn.Process
Simple chemical	The simple chemical shape	com.yworks.sbgn.SimpleChemical
Source sink	The source/Sink shape	com.yworks.sbgn.EmptySet
State variable	The state variable shape	com.yworks.sbgn.StateVariable
Submap	The submap shape	com.yworks.sbgn.Submap
(Left-oriented) tag	The (left-oriented) tag shape	com.yworks.sbgn.Tag
(Right-oriented) tag	The (right-oriented) tag shape	com.yworks.sbgn.Tag with the com.yworks.sbgn.style.inverse attribute set to true
Unit of information	The unit of information shape	com.yworks.sbgn.UnitOfInformation
Unspecified entity	The unspecified entity	com.yworks.sbgn.UnspecifiedEntity
Uncertain process	The uncertain process shape	A regular process shape with a question mark as its label
Catalysis	The catalysis edge	A line with the end equal to an empty circle
Consumption	The consumption edge	A simple line
Inhibition	The inhibition edge	A line with the end equal to a T-shape
Modulation	The modulation edge	An arrow with the head equal to an empty diamond
Necessary stimulation	The necessary stimulation edge	An arrow with the head equal to an empty triangle and a bar
Production	The production edge	A regular arrow
Stimulation	The stimulation edge	An arrow with the head equal to an empty triangle

To facilitate the use of the yEd editor for developing SBGN diagrams, a converter to connect the yEd-specific GraphML and the SBGN-ML formats was needed. Converters exist for SBGN-ML from/to other formats [8] including systems biology standard formats such as Systems Biology Markup Language (SBML) [9], Biological Pathway Exchange (BioPAX) [10], or the SBML-specific to the CellDesigner platform [11]. The work described here presents the ySBGN dual converter that handles the bidirectional translation between the yEd-specific GraphML and the SBGN-ML Process Description formats for metabolic networks.

## 2 Methods

### 2.1 Implementation

The ySBGN converter is a standalone Java-based framework that uses (i) the libSBGN library [12] to manage SBGN resources and (ii) the jgrapht-core library (http://jgrapht.org/) to manage the SBGN graph and export it to the yEd-specific GraphML format. Specifically, the SBGN-to-yEd conversion takes as input an SBGN PD file with a metabolic network and generates the corresponding GraphML file to be managed in the yEd framework. Information about SBGN glyphs and arcs from the input file is stored in a DirectedGraph object (through the jgrapht-core library) that is further used to map the content to the yEd-specific GraphML output file. For the yEd-to-SBGN conversion, the content from the yEd-specific GraphML file is read and parsed to map the structure of an SBGN object (through the libSBGN library), which is used to export the SBGN-ML file; the output file can be further managed using SBGN-specific tools, e.g. SBGN-ED [13]. The ySBGN converter provides a graphical user interface and files can be converted one by one or in bulk. Detailed information on elements such as colour and annotations is conserved during both translations and stored using (i) SBGN-ML extensions in SBGN files and (ii) shape attributes for the yEd-specific GraphML files.

For most elements of the metabolic networks, the conversion was direct due to the yEd SBGN Palette which closely follows the SBGN PD specification (see Table 1). The converter also handles the representation of SBGN elements not available in the yEd SBGN Palette (e.g., clone marker, the association and dissociation processes – see Section 2.3). However, the challenging task was to manage ports of the process glyphs. Here, we briefly outline the solutions in these two cases.

### 2.2 Representing the process glyph in yEd

#### 2.2.1 Conversion from SBGN-ML to yEd-specific GraphML

**Figure 1:**
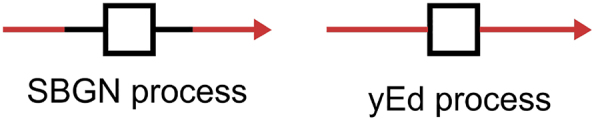
The SBGN process glyph versus the yEd process glyph. In black there are represented the process glyph (square box) and the ports (black line). In red there are represented the incoming and outgoing arcs. The SBGN process glyph connects to these arcs through the ports which is represented by the two black lines on each side of the square box. The representation of yEd of a process does not consider ports by default: The incoming and outgoing arcs are directly connected to the process square box.

In SBGN PD, a process glyph is represented by a square shape with two connected arcs (“antennas”) attached to the centres of opposite sides [3], (as shown in Figure 1). These two connected arcs are referred to as “ports” in the SBGN-ML and correspond to the location where the incoming and outgoing edges connect to the process glyph. This detail is highly important to correctly depict the directions of biological processes and separate two sides of the reaction equation, especially in the case of reversible reactions. Since the shape is very specific and is not available in the yEd SBGN Palette yet, we introduce automatically additional bend points to visualise process ports in yEd.

#### 2.2.2 Conversion from yEd-specific GraphML to SBGN

Before performing the arc-port assignment, there are the following two major features related to the type and the number of arcs connected to ports to be considered in order to obtain correct representations of the molecular processes in SBGN [3]:

1. arcs connected to the same port must have the same type;
2. each port of a process must be connected to at least one arc.

**Figure 2:**
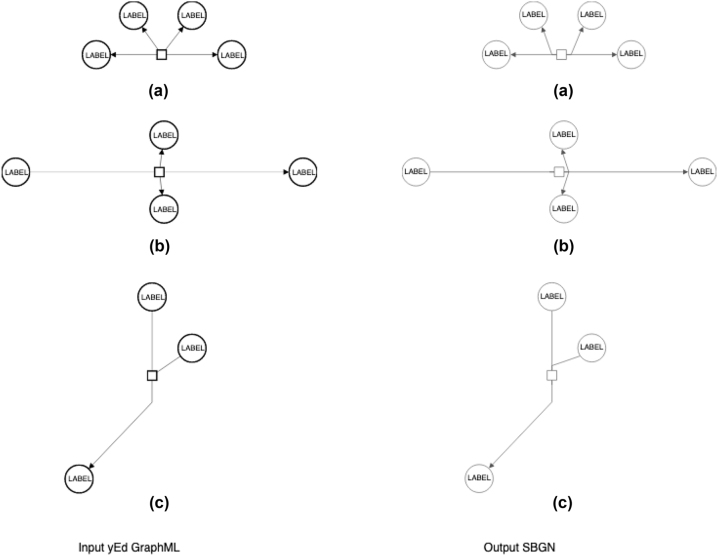
Examples of the conversion of processes in the ySBGN converter: (a) Reversible process; (b) irreversible process with horizontal orientation; (c) irreversible process with vertical orientation.

These features are of particular interest in combination with the reversibility property of a process, as follows:

- For the *irreversible* processes, where at least one arc connected to the process glyph is of the Consumption type and another of the Production type (Figure 2b and c), each process port must be connected to a single type of arcs to achieve feature (1) (above).
- For the *reversible* processes, where all arcs connected to the specific process glyph are of the same type, i.e. of the Production type (Figure 2a), the feature (1) (above) is achieved by default; however, the algorithm must check that each port has arcs connected – to achieve feature 2 (above).

More, (i) the “arc position” (defined here by the endpoint or the nearest bend point, on the left/right/up/bottom side from the process glyph) and (ii) the process orientation should be considered to reduce the number of arcs crossing, as well as, to increase the arc symmetry and to achieve the arc length uniformity in the process representation; these are aesthetic requirements in editing molecular diagrams (see [7] and references therein). The process orientation can be “vertical” or “horizontal” and is encoded in the “orientation” tag [3].

We developed an algorithm to automatically create the process glyphs with ports and to identify and connect arcs to the corresponding ports. Initially, all processes are considered to be horizontally oriented; this is corrected, if needed, in Step 3 (below). The algorithm first detects the sets of (i) irreversible and (ii) reversible processes:

Step 1. In the case of the irreversible processes (a), the algorithm connects the Consumption type arcs to one process port and the Production type arcs to another. In this way, each process port is connected to a single type of arcs.

Step 2. In the case of reversible processes (b), arcs are assigned to process ports based on the Euclidian distance between the arc position and the given process glyph. Once all arcs are assigned, the algorithm checks first if each port has any connected arcs because, in the situation where all connected arcs were closer to one port, they all were assigned initially to that port, meaning that the second port has zero ports connected. Specifically, arcs keep information on their connected glyphs in the Source/Target fields. When an arc is connected to a process port, its source or its target is given by the process port itself. For checking if each of the process port has at least one arc connected, the algorithm verifies if the port itself corresponds to the source or the target of at least one arc. In case one port has no arcs connected, the algorithm re-assigns the arc located at the greatest distance (farthest) from the initially connected port to another port, assuring that each port of a process is connected to at least one arc.

Step 3. Further, the process orientation is calculated based on the position of the connected arcs. If most of the arcs are in the left/right sides of the process glyph, the orientation is set to horizontal (Figure 2a and b); otherwise, if the arc majority is found on the top/down sides of the process glyph, it is set to “vertical” (Figure 2b).

### 2.3 Managing SBGN elements without direct representation in the yEd SBGN Palette

*The association/dissociation processes*, which have no direct representation in the yEd SBGN Palette, are currently shown as generic processes by the ySBGN converter in the generated yEd-specific GraphML file.

*Clone marker*: the ySBGN converter integrates an algorithm that was implemented for detection of clone elements in the diagram to represent them in the generated SBGN file.

*Up/Down tags* are translated to left-oriented tags by the converter due to the fact that currently there are no up/down-oriented tags in the yEd SBGN Palette. A simple test case is given in the tags folder (available at https://github.com/sbgn/ySBGN/wiki/Limitations).

### 2.4 Conversion time

For the same files, the time for the yEd-to-SBGN conversion is higher than for the SBGN-to-yEd direction due to the execution of the detection algorithms for process ports and clone markers. During the yEd-to-SBGN conversion, an algorithm is also run to parse and connect the auxiliary units (state variable, units of information) to corresponding glyphs, given the fact that the yEd models store such information as elements that are referred by identifiers from the corresponding glyphs rather than as internal glyphs like in SBGN. The difference in the conversion time between the two directions depends therefore by the size of the input files and of the set of elements without direct representation in the yEd SBGN Palette.

## 3 Results and discussions

### 3.1 ReconMap in the yEd-specific GraphML format

Of particular interest is achieving the translation of the ReconMap resource [14] to the yEd-specific GraphML format. The ReconMap resource is the most comprehensive, highly curated and manually drawn map (with 3763 unique species and 5535 metabolic reactions) based on the Recon2 human metabolic reconstruction [15].

**Figure 3:**
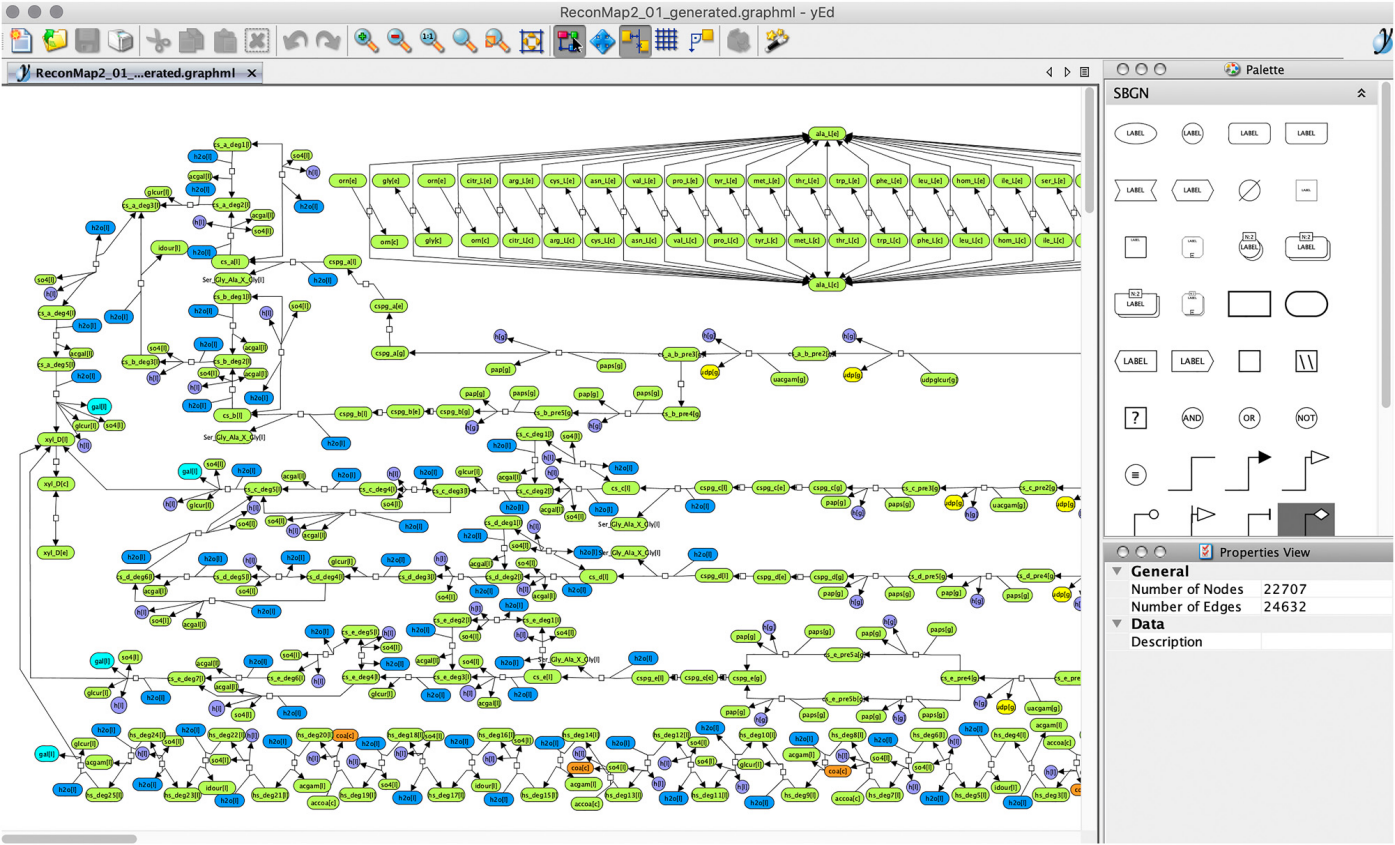
The ReconMap 2 in the yEd Graph Editor. The ySBGN converter translated the ReconMap resource available in SBGN-ML format into the yEd-specific GraphML format. Information on colours and annotations is also preserved during the translation. Please note the SBGN Palette on the right side, available since the yEd 3.17.1 release.

The ySBGN converter imported the SBGN-ML version of the ReconMap diagram1The ReconMap resource, initially available in CellDesigner xml format at https://www.vmh.life, was converted to SBGN-ML format by the cd2sbgnml tool [16], a complementary tool that converts between CellDesigner-specific SBML extension and SBGN-ML. and translated it to the yEd-specific GraphML format to facilitate management (visualisation, modification) of the content using the features of the yEd editor. Figure 3 displays the ReconMap (GraphML) in the yEd editor following conversion using the ySBGN converter. The converter also enabled the translation of the yEd-specific GraphML ReconMap file to SBGN-ML to permit users storing the content in the SBGN-ML standard format in case they updated the diagram while using the yEd editor.

#### 3.1.1 Conversion time

The conversion was performed using a MacBook Pro (3 GHz Intel Core i7, 16 GB) and took 96 s from SBGN to yEd-specific GraphML and 1010 s inverse. The difference between the timing is due to several additional steps that are made during the yEd-to-SBGN conversion to generate a valid SBGN file, such as the algorithms for identification of (i) the process ports and connected arcs, (ii) the clone marker and (iii) the auxiliary units and corresponding decorated glyphs (see *Implementation*).

#### 3.1.2 Availability of generated files

The generated GraphML and SBGN-ML files for the ReconMap2_01 resource are available under the GPLv2 licence at https://github.com/sbgn/ySBGN/wiki/Examples#ReconMap2_01. In addition, the yEd-specific GraphML generated files for several metabolic pathways taken from a library of the SBGN examples (https://github.com/sbgn/libsbgn/tree/master/example-files) are available in the GitHub Wiki (https://github.com/sbgn/ySBGN/wiki/Examples).

## 4 Future work directions

Several limitations that have been identified during the use of the ySBGN converter together with corresponding further development steps to solve them are presented briefly below. An updated list of encountered limitations is available on the ySBGN-dedicated github repository at https://github.com/sbgn/ySBGN/issues.

First, the ySBGN converter currently uses a graph reconstruction for translation between the two XML-based files (stored yEd-specific GraphML and SBGN-ML formats), which is a complex and time-consuming process. The implementation of an XSLT-based approach, which aims to create an XSLT stylesheet to allow importing XML files to the yEd-specific GraphML, is planned for the converter’s code. This approach would be more flexible to accommodate updates required by potential modifications in the formats (such as changes in the structure, addition of more complex glyphs).

Another limitation is the fact that the conversion hangs after an exception is thrown. Therefore, the development of a log system to record and inform the user on warnings and errors during the translation is also planned. The log system will capture and notify all types of activities, including (a) simple notifications e.g. changes made by the converter to accommodate conversion of SBGN elements without direct representations in yEd SBGN Palette (such as the association/dissociation processes and the tag glyphs), (b) warnings e.g. existence of output files in the selected folder and that these will be overwritten during the current conversion and (c) errors e.g. the translation direction being incompatible with the type of the selected input files. Thus, a log system will guide the user and will facilitate the usability of the converter.

In terms of time performance, the conversion of large scale maps takes a considerable amount of time. This is primarily due to the arc-port assignment step that includes several verifications to generate a correct process representation following the SBGN specifications [3]. In order to optimise the conversion time, several steps (including the identification of clone markers and auxiliary units) were identified to run in parallel; an implementation is planned.

Further work includes extending the ySBGN converter to accommodate SBGN diagrams specific to signalling processes, and this requires introduction of new types of entities and types of processes. Since we noticed that in some more complex cases, the port orientation is changed, we will also reevaluate the set of features influencing the assessment of the process orientation focusing on the signalling processes.

The integration of the extended converter into the System Biology Format Converter (SBFC) [8] and development of a validation step of the generated SBGN map (similar to e.g. SBGN-ED [13]) are also planned. Finally, the aim is to integrate and provide the ySBGN functionality as a web-based application in order to improve the user’s access.

## 5 Conclusions

The ySBGN converter permits translation between the yEd-specific GraphML and SBGN-ML formats, allowing management of SBGN diagrams by the yEd framework, a well-established graph editor that can support extensive and dense graphs. Using the yEd platform substantially reduces the amount of work for systems biology researchers when describing a model: an aesthetical view is created easily with a possibility of having the network represented and visualised in the standard format.
